# Road Transport: A Review of Its Effects on the Welfare of Piglets

**DOI:** 10.3390/ani13101604

**Published:** 2023-05-11

**Authors:** Martina Zappaterra, Luigi Faucitano, Leonardo Nanni Costa

**Affiliations:** 1 Department of Agricultural and Food Sciences (DISTAL), Alma Mater Studiorum University of Bologna, Viale Fanin 46, I-40127 Bologna, Italy; leonardo.nannicosta@unibo.it; 2 Agriculture and Agri-Food Canada (AAFC), Sherbrooke Research and Development Centre, Sherbrooke, QC J1M 0C8, Canada; luigi.faucitano@agr.gc.ca

**Keywords:** animal welfare, behaviour, loading density, mortality, piglets, stress, transport duration, weaning

## Abstract

**Simple Summary:**

An extensive body of literature has been produced on the effects of transport-related factors on the welfare of adult pigs, while less attention has been paid to weaned piglets. Understanding how to safely transport weaned piglets is very important as they present their own set of challenges and risks. Despite this gap in the scientific literature produced to date, transporting piglets immediately after weaning is a common practice and every year millions of weaned piglets are transported on long journeys to reach the growth-finishing farms. The combination of the stress of weaning and transport can lead to negative effects on the health and welfare of piglets. The review will provide an in-depth discussion of the main factors that affect the welfare of weaned piglets during transport and will highlight the need for more research on key factors affecting the welfare of piglets during transport aimed at providing scientific evidence to the existing recommendations and/or regulations.

**Abstract:**

The purpose of this review is to present the best available scientific knowledge on key effects of pre-transport and transport factors influencing the response of piglets to transport stress and post-transport recovery. To date, research on piglet transportation particularly focused on the effects of season (i.e., heat and cold stress), vehicle design features (ventilation type and deck/compartment location), space allowance and transport duration, and piglet genetics. More specifically, in this review the effect of transport duration has been dealt with through its impact on death rate, behaviour and physiological response, and feeling of hunger and thirst. Based on the available literature, clear conclusions can be drawn on the vulnerability of piglets to heat stress during transport. Both short and long transportation have an effect on piglet welfare, with effects being biased by the genetic background, ambient conditions and vehicle design. Further studies investigating the impact of factors such as vehicle design, truck stocking density and environment, piglet genetic background, and weaning age are needed.

## 1. Introduction

Pigs are transported for sanitary and market reasons throughout their lives, from weaning to slaughter [1]. Transport is a multi-factorial stressful event where a combination of factors, i.e., vehicle design, ambient conditions (temperature, humidity, vibrations, and noise), journey conditions (duration, space allowance, and driving quality), and handling at loading and unloading, rather than a single one is responsible for the animal’s well-being [2,3].

Although the transport of pigs has largely been investigated over the past years, most studies have focused on finishing pigs destined for the abattoir, while less attention was addressed to newly weaned piglets and weaners [4,5]. The first transports which happen in pigs’ life actually take place soon after weaning, when newly weaned pigs are transferred for a short distance to nursery barns, while weaners (around 30 kg) are transported over short or long distances from the nursery barn to reach a different farm where they will be reared until they reach a market weight [1,6]. Newly weaned piglets are mainly transported for biosecurity reasons, with the aim of relocating them to a different site, reducing the risk of transmission of infectious agents. On the other hand, weaners are usually transported as sow herds, which are located in more remote, biosecure regions, while grow-to-finish barns are located near feed production and slaughter facilities [5]. In both pig types, transport immediately after weaning can exacerbate the negative effects of weaning stress due to prolonged fasting, as well as increasing the risk of diarrhea and delayed growth [7,8,9].

Weaning and related practices represent a particularly stressful phase in the life of pigs [10] as they strongly affect the metabolic state of the animals, triggering in some cases disruptions in intestinal barrier function and permeability [10,11]. Disruption in the homeostasis of the intestinal mucosa can lead to the translocation of bacteria, antigens and toxins to other tissues, leading to states of inflammation, and, in severe cases, bacterial infections. Due to the diet changes and stress associated with this phase, weaning can also lead to energy, lipid and protein metabolism dysfunctions, affecting kidney and liver functions [12]. Weaning is a stressful phase from a social perspective too as the piglet is separated from the mother and littermates and mixed with unfamiliar individuals. This social stress can have effects on its behavioural development as well as increasing cortisol production, the main stress-induced glucocorticoid, with effects on innate and adaptive immunity [13]. In addition, piglets are growing animals with immature intestinal mucosa, which puts them at greater risk of developing disease states. This deadly cocktail of factors makes weaning one of the most critical periods in pig management.

According to EU Council Directive 2008/120/EC [14], in the European Union, no piglets shall be weaned from the sow at less than 28 days of age, unless the welfare or health of the dam or piglets would otherwise be adversely affected. In practice, in most commercial pig farms, weaning is anticipated to be at three weeks of age. Intra-EU trade of piglets involves more than 15 million heads per year, with exports mainly from Denmark and The Netherlands and mostly heading to Germany, Poland, Belgium, Spain and Italy [15]. In the intra-EU trade, distances between the origins and destinations are generally within 24 h of travel as the countries exporting and importing piglets are predominantly those in central Europe, as well as Denmark, France and the northern regions of Spain and Italy [1]. Despite the relevance of this trade to the EU, studies on the effect of transport on piglets have been mainly carried out in Canada and USA, where piglets, also called Iso-Wean piglets, are weaned around 17 days after birth and the transport occurs immediately after weaning [7,8,9,16,17,18].

Despite these differences between countries in age at weaning, transporting piglets immediately after weaning is a common practice. However, the combination of the stress of weaning and transport can lead to negative effects on the health and welfare of piglets [19]. The present review aims at analysing and discussing the main factors that affect the welfare of weaned piglets during transport and their post-transport recovery at the farm of destination.

## 2. Materials and Methods

**Figure 1:**
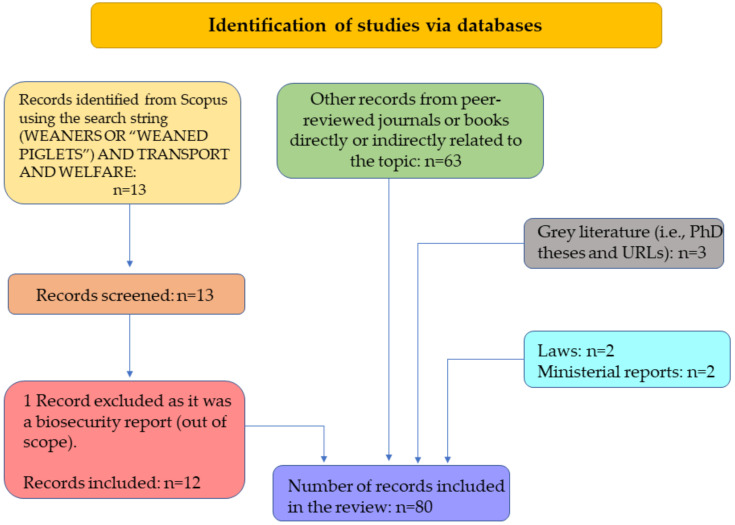
Literature search flow diagram following PRISMA guidelines. The number of studies and documents that were found, retained, and further considered are shown at each stage of the literature review process.

The literature search was performed using Scopus^®^ database (www.scopus.com) in November 2022 and then repeated in February 2023. The used search terms were (WEANERS OR “WEANED PIGLETS”) AND TRANSPORT AND WELFARE. Given the limited number of articles being found (only 13 peer-reviewed publications), no limitation was set in the time span of the search. Grey literature was also searched using Google Scholar database based on the same search terms. An additional assessment of bibliographical items resulted in further identification of relevant sources of information, which were then included among the list of documents of interest. The bibliographic material—mostly peer-reviewed resources and in some instances government publications—was restricted to primarily English language and non-English publications which included an English abstract, where relevant information could be retrieved. Once relevant publications were identified, they were filed and categorized for further evaluation. Figure 1 shows the number of studies and documents found and retained at each step of the literature review process.

## 3. Before Departure from the Farm: Weaners’ Health and Management

### Animals’ Health Status and Fitness for Transport: An Ounce of Prevention Is Worth A Pound of Cure!

Quoting Grandin, “it is impossible to assure good animal welfare during transport if the animal is unfit” [20]. Fitness for transport is defined as the animal’s ability to withstand transportation without compromising its welfare [21]. A proper assessment of fitness for transport is essential to prevent suffering in animals and avoid deaths during transport or in the hours following the arrival [21]. Poor fitness for transport was the major source of the high DOA rate (0.3%) reported by Valkova et al. [22] based on the analysis of a 10-years retrospective database of piglet transports in the Czech Republic. EU Council Regulation (EC) No 1/2005 mandates that piglets must not be transported if they are new-born with yet unhealed navels, if they present severe wounds or prolapses, if they are unable to move or walk unaided and without pain, or are aged less than three weeks. In the latter case, piglets aged less than three weeks must only be transported less than 100 km [23]. Other health conditions that may make a piglet unfit for transport include the presence of respiratory distress [24]. Therefore, pigs showing lameness, hernias, injuries and wounds, abnormal discharges, diarrhea, and breathing difficulties need to be carefully inspected before being transported as, based on the severity of these states, their ability to cope with transport-related stressors may be impaired [24].

Among these clinical signs, hernias (i.e., the common term used for umbilical outpouchings) are commonly observed in piglets [25]. Umbilical outpouchings can be a clinical sign of various abnormalities, which can range from the less serious (cysts or mild hernias) to more problematic states, such as abscesses and severe hernias that can become strangulated. Depending on the size, location, and underlying pathology, umbilical outpouching can be considered a problem that hinders the normal mobility of pigs and their ability to balance during transport practices [26]. In addition, vibrations and possible loss of balance during on-road transport could cause the rupture of abscesses and cysts, or in cases of serious hernias, their strangulation or rupture. An association between presence of hernias at departure from the farm and increased mortality (×1.3) has been reported in a survey of adult pigs transportation [27]. Unfortunately, there are no data on the incidence of hernias and related transport mortality in piglets.

Depending on the breed and sex, umbilical hernia affects between 1.6 and 7.8% of piglets [28]. Increased incidence of hernias may be due to several genetic and environmental factors, including abnormal behavioural patterns among piglets, such as belly nosing and navel sucking [29]. Herniated animals may be an even more attractive target for the perpetrators of belly nosing and suckling, exposing such animals to increased risks during travel and in the days immediately following the start of weaning, when these behaviours tend to peak [30].

Prolapses are mentioned in EU Council Regulation (EC) No 1/2005 as a factor that makes an animal unfit for transport [23]. However, there are no real indications on the size beyond which a hernia should be considered dangerous. According to the guidelines published by the Consortium of the Animal Transport Guides Project [24], pigs are unfit for transport if they show umbilical outpouchings wider than 15–20 cm and with sores. However, besides not having been validated for its effect on the welfare of pigs during transport by any controlled study, it is not specified whether this recommendation for maximum size applies to all pig ages and categories, or whether it is only applicable to adult pigs. Given the smaller size of piglets when compared with adult individuals, it is indeed reasonable to assume that the limits for severe umbilical outpouching size should be lower in piglets.

Traumatic injuries and bruises may be among the factors that make a piglet unfit for transport, especially if they result in obvious blood loss or a state of pain during limb movement. Limb lesions seem to be quite frequent in piglets, with a high prevalence of limb abrasions and sole bruises and erosion resulting from their housing on hard concrete floors [31]. Valkova et al. [32] evaluated the prevalence of traumatic injuries on the carcasses of piglets sent to slaughter and reported a higher prevalence of limb injuries in piglets than finisher pigs (0.15 vs. 0.10%). However, this difference could also be related to the fact that piglets are not a pig category normally slaughtered in the Czech Republic [32,33], and therefore those sent to slaughter are culled animals that were excluded from the growth phase due to health problems and slower-than-normal growth performance. Although most injuries and bruises may not be serious, piglets with limb lesions may be less able to balance themselves during transport and thus more prone to slips and falls resulting in injuries and lesions that can get infected and possibly turn into abscesses and bursitis.

Other clinical conditions that could adversely affect the welfare of piglets during transport include pathological states, such as diarrhea and weakness. Emaciated and weak piglets may not be able to survive stress, feed deprivation and dehydration that occur during the first hours of the weaning process and during transport. Apart from the information taken from recommended practices for adult animals [24], the lack of scientific evidence does not allow us to associate the incidence of transport-related losses with different body and health conditions and their monitoring at the departure from the farm in piglets. Therefore, it is reasonable to think that the mortality observed during the weaning stage may in some cases be the result of the additive effect of weaning and transport of likely unfit piglets.

The genetic background can also contribute to the response to transport stress in piglets. A transport study showed that piglets heterozygous for the *Ryanodine Receptor 1* (*RYR1*) or *Halothane* (*HAL*) gene (i.e., piglets displaying the TC genotype, alias HAL^Nn^, for the C1843T RYR1 mutation) presented a greater physiological response to transport, as shown by the increased blood albumin concentrations and total white blood cell and neutrophil counts, when compared with the CC homozygous (alias HAL^NN^) [34].

Although they are not science-based, the recommendations or regulations for the monitoring of fitness for transport must be also applied to piglets. Sick, herniated, or emaciated piglets should not be transported, but should be kept, when possible, in the herd of origin to be treated and left to heal before being shipped to the growing site.

## 4. Factors Influencing Piglets’ Response to On-Road Transport

### 4.1. Environmental Conditions during Transport

#### 4.1.1. Thermoregulation Principles

Pigs are homeotherm mammals, and thus, like other homeotherm animals, produce several physiological, endocrinological, and behavioural responses to thermoregulate and maintain constant their core body temperature. A stable core temperature can be maintained only when heat production and heat loss are balanced [35]. Thermoregulation responses vary depending on how far the ambient, skin, and body core temperatures are from the animal’s biophysical requirements. Thermal balance is indeed dependent on a combination of these temperatures, and the animal’s tolerance to different ambient temperature ranges which varies by age, sex, and breed. Thermal homeokinesis is a steady state where the internal body temperature of a homeotherm animal is kept constant at the normal core temperature level with little additional energy expenditure [35]. Thermal homeokinesis is maintained when ambient temperatures and environmental parameters are within the range of Thermal Comfort Zone (TCZ). The concept of TCZ was first hypothesized based on the human perception of the thermal environment [35] but is now also applied to livestock species. Translated into animal welfare, the TCZ is the range of environmental parameters (mainly temperature) where the energetic and physiological efforts for thermoregulation are minimal and within which an animal expresses satisfaction with the thermal environment, and does not need to change its behaviour to cope with the environment [35,36,37]. Outside the TCZ, the animal starts to experience thermal discomfort, which drives thermal-related behaviours (e.g., huddling, posture adjustments, searching for shaded places, etc.) that anticipate autonomic thermoregulatory mechanisms [35,36,37]. The TCZ is comprised within a wider range of ambient temperatures, namely the Thermoneutral Zone (TNZ), which is defined as “the range of ambient temperature at which temperature regulation is achieved only by control of sensible (dry) heat loss, i.e., without regulatory changes in metabolic heat production or evaporative heat loss” [38]. The TNZ boundaries are represented by the Lower Critical Temperature (LCT and the Upper Critical Temperature (UCT). Temperatures (absolute or perceived) below the LCT lead to cold stress in homeotherm animals. Below LCT, metabolic heat production increases, as the animal attempts to keep body core temperature in an acceptable range using shivering (irregular frequent muscle contractions), vasoconstriction, and in some animal species also by activating brown adipose tissue catabolism [39]. When environmental temperature and humidity values are below the LCT and the physiological responses activated by the body are not able to maintain or restore an acceptable core temperature, the animal enters hypothermia. Hypothermia is associated with several organ failures, cardiovascular dysrhythmias, such as ventricular fibrillation and pulmonary edema. The central nervous system’s electrical activity is also noticeably diminished [40] and, when hypothermia is severe and prolonged, multiple organ failure and death may occur [41].

Temperatures (absolute or perceived) above the UCT lead to heat stress in homeotherm animals. At this point, physiological, endocrinological and behavioural responses are activated to counteract the increase in core body temperature. The energy expenditure to activate these responses increases; thermoregulation is mainly sought with increased water evaporation from the body surface (thermal sweating) or the respiratory ways mucosa (thermal panting) [42]. These responses are often combined with other endocrinological and physiological processes aimed at maintaining a stable core body temperature. When heat stress is prolonged over time, animals experience health issues, infertility, decreased growth and production, decreased immune system efficiency, and cellular and mitochondrial oxidative damage [43]. When the body’s ability to thermoregulate becomes disrupted, overheating (hyperthermia) and, in the most serious cases, organ failure and death may occur [41].

#### 4.1.2. Piglet Thermal Needs during Transport

EU Council Regulation (EC) No 1/2005 mandates that vehicle interior temperatures during animal transport must remain between 5 °C and 30 °C, with an acceptable variation of ±5 °C. It also states (Chapter 2) that transported animals must be protected from extreme temperatures and must not suffer [23]. However, the acceptable range of temperatures shown in this law is not sufficient to ensure the protection of all animals from suffering and extreme environmental conditions as it does not consider the differences in the perception of TNZ between animal species, determined by species categories, age, weight, sex, genetics and rusticity [44,45].

In pigs, the science-based recommended upper limit of TNZ is considerably lower than that indicated by the current EU regulation. Depending on the pig categories, UCT appears to vary between 19 °C for boars and 30 °C for piglets (8–30 kg liveweight [26,44]). However, such ranges have been observed in pigs kept on farms, whereas very little work has been done to establish the TNZ ranges of weaners during transport.

As mentioned in the previous paragraph, both temperatures below the LCT and above the UCT can jeopardize the welfare conditions of pigs. While in market and heavier pigs (such as sows and boars) high temperatures pose a serious welfare risk during transport, piglets have a better tolerance to higher temperatures, having a range of TNZ more shifted towards higher temperatures [26,44]. This is related to the fact that these young individuals still do not have a thick subcutaneous fat layer that can impair their heat-dissipating capacity, and have a greater surface-to-body mass ratio when compared to adult individuals [46,47]. Furthermore, unlike other animal species, piglets do not possess brown adipocytes [47,48], which are functional in preventing heat dissipation (non-shivering thermogenesis), and therefore their thermoregulation relies exclusively on huddling behaviours and shivering when exposed to cold temperatures [48].

Most publications reporting the effects of ambient temperature on the behaviour and physiology during transport, and post-transport performance of piglets were produced between 2005 and 2014 [16,18,49,50,51,52,53]. In the study by Lewis et al. [53], early-weaned piglets (17 ± 1 days of age) were transported for 0 h, 6 h, 12 h or 24 h during three seasons, summer (22.1 °C to 30.3 °C), fall (3.4 °C to 12.8 °C) and winter (−2.8 °C to 3.2 °C). Ear skin and rectal temperatures during transit were higher during the summer than during fall or winter. No significant effect of season was found on ear skin temperature, weight loss or average daily gain during the first 7 days and on day 14 post-transport. However, piglets transported in winter had lower rectal temperatures during 12–24 h transports, suggesting that they were unable to compensate when temperatures inside the truck dropped for longer periods, such as in transports lasting between 12 and 24 h. The cold stress experienced by these piglets during the winter journeys was also confirmed by the reduced social interactions between conspecifics and the increased frequency of resting behaviour during the journey reported in a previous study [16]. In a similar study, Wamnes et al. [51] found that early-weaned pigs transported for 6, 12 or 24 h in winter (within vehicle temperature ranging from −4 °C to +14 °C) had greater weight loss and lower average daily gain than piglets transported in summer (within vehicle temperature ranging from 14 °C to 25 °C) during the first 8 days and on days 10, 12 and 14 post-transport. This increased weight loss was explained by the incremented energy expenditure and muscular activity (with shivering) performed to maintain homeostasis. However, even transport during summer appeared to affect the welfare of piglets as, during the first 4 days after transports of 6, 12 and 24 h, they spent more time lying and showed longer drinking bouts than piglets transported in winter or not transported [51,52]. These results may suggest that journeys longer than 6 h at temperatures around 25 °C may lead to exhaustion and dehydration in piglets, probably caused by an increased amount of energy spent to increase heat loss through panting. The effect of season on the behaviour, physiology and mortality rate of transported weaned piglets has also been investigated in other studies [6,8,18,49,50]. However, due to the experimental design applied in these studies, the effects of season were biased by the additive effect of transport duration.

A recent study has also shown the effect of higher temperature-humidity index (THI) values (THI > 85) on behavioural and physiological parameters in piglets of about 25 kg liveweight (68 days old) transported in a double-decked, open/passively-ventilated vehicle [54]. A difference in THI values was recorded between decks, with the bottom deck presenting higher THI values compared with the top deck (>83 vs. <82). In this study, relative humidity (RH) more than temperature, which did not differ between decks, contributed to THI variation. Piglets transported in the bottom compartments (THI: approx. 86) showed higher mean rectal temperatures and respiratory rate, as well as greater blood cortisol concentrations, when compared with piglets transported in the top deck compartments. These signs of thermoregulation issues (starting from THI > 85) may be explained by the poor ventilation rate within the bottom deck resulting in the production of an oversaturated atmosphere (RH > 80%) in this vehicle location. Based on the results obtained under the experimental conditions of this study, a UCT of 30 °C with a maximum RH of 80% were finally recommended to ensure the thermal comfort of piglets during transport [54].

The scientific literature regarding the effects of different temperature and RH values on the welfare of piglets during transport is particularly scant. Furthermore, since thermal needs are closely dependent on the weight and growth stage of the animals, the differences in weaning age of piglets between studies make the identification of TNZ values for this pig type even more complex. The almost total lack of comparative studies between different breeds and genetic lines also needs to be highlighted. It is indeed reasonable to think that some autochthonous breeds reared in geographical locations with tropical climates are more able to withstand higher temperatures and RH values. To the best of our knowledge, there is only one study that compared heat stress resistance (based on respiratory rate and rectal temperature) between Large White and Creole piglets (25 kg liveweight) [55]. Based on the results of this study, Creole piglets had higher UCT values compared with Large White subjects (UCT = up to 34 °C vs. 32 °C, respectively), which confirms the contribution of the genetic component to the heat stress resistance of pigs.

### 4.2. Space Allowance

The microclimate (temperature and RH) inside the vehicle is also closely dependent on other variables related to transport conditions, including loading density (kg/m^2^) or space allowance (m^2^/kg). Research has shown a positive relationship between the size of the load and the within truck temperature that could increase up to 7 °C for every two pigs added to the load [56]. New recommendations for loading density/space allowance calculated following the allometric equation (K-value based on space need for standing/sternal lying, and semi- and fully recumbent position) suggested by Petherick and Phillips [57] have been proposed for the transport of piglets, finishers and sows, gilts and boars in recent EFSA opinion [26].

EU Council Regulation (EC) No 1/2005 [23] establishes that pigs transported by road must be provided with sufficient space to lie down or stand up in their natural position. Depending on the meteorological conditions, the floor surface area per animal may also be increased by a maximum of 20% [23].

While the effect of space allowance on the welfare and carcass and meat quality of slaughter pigs has been largely investigated [3,58,59,60], very little is known about the effect of floor space in the truck on the welfare of transported piglets.

The few available results would indicate a limited effect of space allowance on behaviour and the physiology of weaned piglets during transport. Results from physiological studies range from no effect on blood stress indicators (i.e., cortisol, aspartate aminotransferase, creatine kinase, and glucose concentrations), immune response (i.e., neutrophil chemotaxis and phagocytosis) and performance measures (i.e., body weight gain and lesion scores) [18,50,61], to increased lymphocyte-to-neutrophil ratio (NL) in piglets transported at lower space allowance (0.50 m^2^/pig vs. 0.06 and 0.07 m^2^/pig) during summer [49]. However, this latter effect recorded in one-hour transportation trials would appear to be more closely related to the combined effect of high ambient temperatures (ranging from 26.1 °C to 30.3 °C) and higher stocking density during transport on piglets’ heat stress condition. The effects of space allowance on piglet welfare during transport have been mostly shown in terms of changes of position during transport, with piglets transported at smaller space allowances (0.05 m^2^/pig vs. 0.06 and 0.07 m^2^/pig) standing/rearing more and lying less [18,49,50]. Increased standing/rearing or sitting behaviors may be considered as an indication of competition for space that may be prevented by applying a minimum space allowance of 0.06 m^2^/pig [49].

The few available results show that, unlike adult pigs [3], overcrowding does not particularly affect the welfare of piglets during transport. The reason for this difference may be two-fold. First, in piglets, overcrowding-related increased temperatures may not result in as severe heat stress as in adult pigs, since younger pigs require higher environmental temperatures (higher UCT) to be in thermal comfort (see Section 4.1.2). Second, spatial proximity and lying in full body contact is a common behavior in transported young pigs, which tends to decrease as piglets grow [62].

In agreement with these observations, the minimum floor space requirements suggested for piglets during transport by the recently published EFSA scientific opinion are 0.13, 0.20, and 0.26 m^2^/pig for piglets of 10, 20, and 30 kg live weight, respectively [26]. These minimum space allowance requirements are similar to those indicated for piglets transported by air in EU Council Regulation (EC) No 1/2005 [23].

### 4.3. Transport Duration

EU Council Regulation (EC) No 1/2005 [23] limits journey duration for pigs to a maximum of 8 h, except when several additional requirements for the vehicle and animal’s needs are met (i.e., vehicles have obtained a type II transporter authorisation). All pigs may be transported for a maximum period of 24 h, except for unweaned piglets, which after 9 h of travel must rest for at least one hour before travelling for a maximum of another 9 h. After this journey time, animals must be unloaded, fed and watered and be rested for at least 24 h. Travel duration has been identified as a priority welfare issue for a long time in Europe and North America. Based on the conclusions of a comprehensive literature review of swine transportation, both long and short transports may result in poor welfare outcomes for pigs [5].

Over the years, the impact of transport duration on the welfare of piglets has been assessed through the study of its effects on mortality (dead-on-arrival, DOA) rate, behavioural and physiological responses, hunger, and dehydration/thirst.

#### 4.3.1. Effects on DOAs

Similarly to adult pigs, DOA rate has been used as an indicator of piglets’ welfare during transport, even if the available results do not allow the identification of hazards specifically responsible for it. Averós et al. [6] surveyed the factors affecting the number of weaned piglets found dead after commercial transport from different farms. Information related to 58,682 piglets during 109 journeys in different EU countries was collected at the end of each journey using questionnaires. Piglets had been weaned at 21 to 28 days of age and were transported at 85 to 100 days of age. Overall, the DOA rate was 0.07%, with dead piglets being reported in 13.8% of the loads. The duration of the journey, ranging from 0.3 to 69 h, and the mean outside temperature, ranging from 0 °C to 38 °C, showed a significant interactive effect, with a gradual increase in the predicted number of DOA with increasing journey duration and outside temperature. The provision of drinking water during the journey reduced the number of dead piglets by almost 2.5%, with an estimated mortality rate at 30 °C of about 3% in eight-hour journeys without drinking water available that dropped to about 0.15% in similar journeys where piglets were provided with drinking water [6]. In this study, an interactive effect on DOA rate in piglets was found between transport duration and vehicle design [6]. When piglets were transported for more than 8 h, mechanically ventilated vehicles exponentially decreased the risk of deaths, with an estimated reduced incidence of dead piglets (approx. 1%) compared with 24 h transports at an outside temperature of 30 °C using passively ventilated vehicles (>8%).

Based on data obtained from 78 loads of weaned piglets (79,715 piglets of approx. 6 kg), including outside and within vehicle temperature, travel distance and time, stocking density, and DOA rate by compartment, Harmon et al. [63] reported an effect of the interaction compartment temperature (ranging from −5 °C to 27 °C) × travel time (average of 8.51 h, ranging from 3.4 to 12.3 h) on DOA rate (0.03%, ranging from 0 to 1.11%), which increased with increasing ambient temperature and transport time. However, a difference was observed in the mortality rate among vehicle compartments during the different seasons. In particular, the mortality rate tended to be higher in winter for the piglets transported in the lower vehicle compartments. The situation reversed during the hot season, when the mortality rate tended to be higher in the upper compartments, probably due to the higher internal temperatures [63]. However, the authors cautioned about the meaning of these results given the very small number of mortality events, with only 10 loads out of 78 (13%) presenting dead piglets on arrival.

More recently, Golightly et al. [17] failed to find an association between mortality rate (0.06%) in weaned piglets undergoing shorter or longer duration (<3 h and >30 h, respectively) commercial transport in the summer.

Based on the published research, it therefore appears evident that the DOA rate is mainly determined by the conditions under which piglets are transported more than the duration of the journey. In particular, high ambient temperatures (e.g., 30 °C) associated with the unavailability of drinking water during transport and the use of passively ventilated vehicles appears to be a major contributor to the increased DOA rate in piglets.

#### 4.3.2. Effects on Behavioural and Physiological Response

Lewis and Berry [16] have examined the effects of the season (summer, fall and winter) on the behaviour of early-weaned piglets during and immediately after transports of different duration (0 or control, 6 h, 12 h, or 24 h) and carried out without supplemental heat (in winter), feed and water. Overall, in transit piglets spent more time lying than standing (75.6 vs. 21.6% of the time). As transport time increased, the percentage of time piglets spent standing significantly decreased, while lying time increased. Resting behaviour, in terms of lying in lateral or sternal recumbency, whether asleep or awake, during transport increased with transport time from 59.8% (1–12 h) to 91.5% (13–24 h), while standing behavior decreased from 36% (first 12 h period) to 7.4% (second 12 h period). The authors speculated that the increased lying behaviour may have been associated with either fatigue or huddling behaviour. The latter assumption may be justified by the fact that this pattern was more defined in winter and fall implying cold as a causal factor. Furthermore, sitting behaviour was more common during the first 12 h of transport (2.8 %) than during the second 12 h of transport (0.3%). Fighting behaviour was infrequent during the first 6 h of transport, but increased significantly in summer compared with winter, which may indicate that the establishment of the dominance hierarchy may have been reduced or halted in the colder transport environment. In this study, it was also shown as an effect of transport itself on piglet fatigue on arrival at the farm, with higher levels of post-transport resting in transported (81.4%) compared to non-transported (control) piglets (77.5%). Such effect was exacerbated by the time in transit as the percentage of time spent resting on the day following the arrival increased after 6 h (80.2%), 12 h (82.2%) or 24 h (81.9%) of transport when compared to control piglets (77.5%).

Magnani et al. [64] examined the effects of long transport (14 h) and ambient temperature and humidity conditions on behaviour and blood parameters of post-weaned piglets (8–12 kg liveweight, approx. 35 days of age). The journey time was divided into three periods of about 4 h each. Similar to Lewis and Berry [16], resting (lying on the belly or in the lateral position with or without head movements) increased with transport duration and, in particular, during the last period of the journey. The increased resting behaviour over time can be considered an indicator of the progressive habituation of piglets to the transport environment. However, this adaptation was not observed in the weaned piglets transported at THI > 72, which rather tended to keep a standing position during the journey, probably in an attempt to dissipate more body heat [64].

Garcia et al. [65] compared unweaned piglets (control) with piglets undergoing weaning (two groups, with and without water and feed for 32 h) and newly weaned piglets transported for 32 h with or without the provision of feed and water. Piglet behaviour was recorded for all treatment groups during transport and 24 h after transport. During the 32 h journey, weaned piglets transported with or without the provision of feed and water spent 77% and 81% of their time lying, respectively, while the control group only spent 65% of their time lying. Untransported weaned piglets provided or not provided with feed and water spent 81% and 87% of their time in the lying posture, respectively. Immediately after transport, newly weaned piglets transported without access to feed and water spent less time lying (20%) compared to weaned piglets that were not transported with free access to feed and water (79% of those piglets were lying) [65]. Overall, the results of this study showed that transportation and weaning have a negative effect on behavior of pigs, especially when feed and water are not provided during transport [65,66].

Both short and long transport duration have an impact on the physiological response of piglets to transportation. Averós et al. [34] studied the effect of journey duration (0.6 vs. 8.3 h) on stress levels as assessed by the analysis of blood creatine kinase (CK) and lacto-dehydrogenase (LDH) activities, both indicators of physical fatigue, in 136 weaned piglets transported from a nursery to a growing-finishing farm. In this study, blood CK and LDH activities increased, particularly after the short journeys. Similar results after transport were recorded by Golightly et al. [17] who found that piglets transported for a shorter duration (<3 vs. >30 h) presented increased blood CK levels, although the values were within the normal physiological range for pigs, and greater serum cortisol and N:L ratios.

In a more recent study, Golightly et al. [67], when comparing two different weaning managements associated with transports of two different durations without food and water, observed that the group of piglets weaned 6 days before being transported for a long time (30 h) were hungrier and thirstier on arrival and lay down less in the pen during the first two post-transport days. In contrast, the other group composed of pigs weaned immediately before short transports (<3 h) lay down more upon arrival at the farm, but also showed more severe ear and body lesions, likely resulting from more intense episodes of aggression. This latter result might have been biased by the different handling of piglets on arrival at the farm, with piglets transported for shorter time being mixed in larger groups (25 pigs) than the other transport group, which was instead housed in smaller group pens (mostly 15 pigs each). There is evidence that fighting rate is greater in larger than smaller groups in pigs [68]. In addition, pigs subjected to short transport showed a lower growth rate during the first two post-transport days when compared to the group of pigs transported for longer time. Besides post-transport fighting activity, this effect could be due to the effect of short transport, whether combined or not with weaning stress. A number of studies showed the effects of shorter transportation on the fatigue condition of piglets on arrival at the farm due to lack of time to recover from the stress of handling at loading and settle and rest completely in the truck [16,34,69].

The effects of longer journeys on blood parameters in piglets are not clear, ranging from no effect on blood CK and N:L ratios after 14 and 32 h, respectively [64,65], to greater blood CK activity in female piglets transported with the provision of feed and water for 32 h [65].

#### 4.3.3. Effects on Hunger

According to the EU Regulation 1/2005, piglets must be provided with only water during the road journey [23]. The fasting period before loading should be carefully planned in relation to the expected length of the journey, to avoid hunger, weight loss, and death [6,23,64]. The recommended fasting period for piglets before transport is 5 h [21]. However, this recommendation should be validated scientifically, as longer fasting periods (up to a maximum of 20/24 h) seem to decrease the risk of DOA in piglets [6].

Berry and Lewis [7] ran two simulated transport trials to study the effects of journey duration (0 h or control, 6 h, 12 h, and 24 h) and ambient temperature (20 °C, 25 °C, 30 °C, and 35 °C) on the post-transport performance of early-weaned piglets. The interaction transport duration × temperature affected liveweight variation during the first 24 h after transport, with piglets either transported for 24 h at high transport temperatures (30 °C and 35 °C) or for 6 h at 20 °C and 35 °C presenting the greatest weight loss in comparison with control groups. Such effects were still present up to 7 days post-transport, indicating the difficulty of young animals to recover from transport stress.

Other Canadian transport studies reported (1) greater weight loss and slower post-transport weight recovery after 20 min transport compared with 6 h transport, likely resulting from a reduced motivation to feed and drink following transport [51], (2) 7.1% weight loss after 24 h of transport with weight loss increasing with increasing transit time [52] and (3) lower body weight on arrival after >30 h compared with <3 h transport (5.6 vs. 6.2 kg), but this difference disappeared after 3–4 days (6.6 vs. 6.3 kg; [17]).

Piglets transported for 32 h without access to feed and water have been reported to lose more body weight (approx. −9%) compared to non-transported piglets with feed and water access [65]. In a similar study, Garcia et al. [66] reported no significant losses in body weight of piglets transported up to 24 h, independently from the availability of feed and water. However, when the journey was extended to 32 h, piglets transported without feed and water or only with feed lost more weight compared to non-transported control piglets and piglets provided with feed and water or only water (−7.4 and −7.5% and −5.7 and −5.5%, respectively).

Information concerning the variation of blood indicators of hunger in weaned piglets during transport is scarce. Studying the effect of short (0.6 h) and long (8.3 h) journey duration on weaned piglets, Averós et al. [34] found a significant decrease in serum glucose concentrations in pigs transported for 8.3 h vs. 0.6 h. Magnani et al. [64] also reported increased blood urea levels as a result of the prolonged fasting in weaned piglets transported for 14 h.

Although feed withdrawal for more than 6 h is associated with decreased blood glucose levels and increased blood free fatty acids (FFA) and urea concentrations, a fasting period prior to transport is highly recommended to decrease the risk of DOA in piglets [6]. Furthermore, the reported results suggest that water withdrawal has a greater effect on weight loss and risk of DOA than feed withdrawal in transported piglets.

#### 4.3.4. Effects on Thirst and Dehydration

The EU Regulation 1/2005 clearly states that pigs may be transported for a maximum period of 24 h, provided they have continuous access to water [23] to prevent thirst and dehydration. Thirst is expressed by increased visits to the drinker, drinking bouts, water intake, and reduced latency to drink after a period of water deprivation. There is no evidence of piglets drinking during transports (14 h; [64]) but increased drinking behaviour in early-weaned piglets after 24 h transport has been reported [16,52].

Elevated total plasma protein and albumin concentrations and hematocrit levels are indicators of dehydration as a result of transport and the associated feed and water deprivation [70]. In most studies, increased blood levels of total plasma protein, albumin and/or hematocrit percentage have been reported after long journeys (from 8.3 to >30 h; [7,17,34]). However, other studies either reported increased blood total plasma protein and albumin concentrations in weaned pigs after a short journey (60 min; [46]) or slight to no variation in blood hematocrit percentage and albumin in weaned piglets transported for 14 and 32 h, respectively, regardless of whether water and feed were provided [64,65].

## 5. Environmental Enrichment in the Truck

Many studies assessed the efficiency of within truck enrichment strategies to improve comfort and decrease stress during transport in market weight pigs [71,72,73]. In piglets, this assessment is only limited to a couple of studies. Roldán-Santiago et al. [74] evaluated several physiometabolic responses to stress in weaned piglets of different age (8, 15 and 22 days) transported at a space allowance of 1.2 m^2^/piglet for 1 h using trucks either with bedded floors or without. Bedding consisted of a 15 cm-thick layer of straw. Regardless of the age, piglets transported without straw bedding showed higher blood hematocrit and glucose concentrations, and decreased blood pH and pO_2_ upon arrival at the farm when compared with piglets transported on straw bedded truck floor. The positive effect of straw may not only be related to the increased comfort around resting, but also to its property as chewable material allowing the expression of rooting [75] and curbing fighting or other negative social interactions [76,77].

A more recent study evaluated the potential positive effect of exposing piglets to music 5 days before transport and during a short transport (approx. 70 min trip) and providing chewable material (toys made with a plastic hose) and lavender aroma (sachets containing 20% lavender) on their skin surface temperature, respiratory rate and behaviour during transport [78]. Music (60 dB maximum sound pressure) consisted of vehicle sounds piglets could listen to at the farm in order to become familiar with the truck environment and pop-rock sound being played at the farm and during transport. Both toys and lavender sachets were hung along each truck compartment side. Overall, enriched piglets showed a lower respiratory rate before transportation compared to control ones. Piglets exposed to vehicle sounds and pop-rock music spent most of their time lying during transport, and were less aggressive and more playful during and after transport, respectively, when compared with piglets subjected to the other treatments. In contrast, an increased mounting behaviour was observed in piglets exposed to the toy and aroma treatments. This behaviour may have been indicative of their attempts to reach the toys and the lavender sachets. However, these results would deserve a further validation under more variable on-farm familiarization/training periods and transport conditions, in terms of duration and climate.

**Figure 2:**
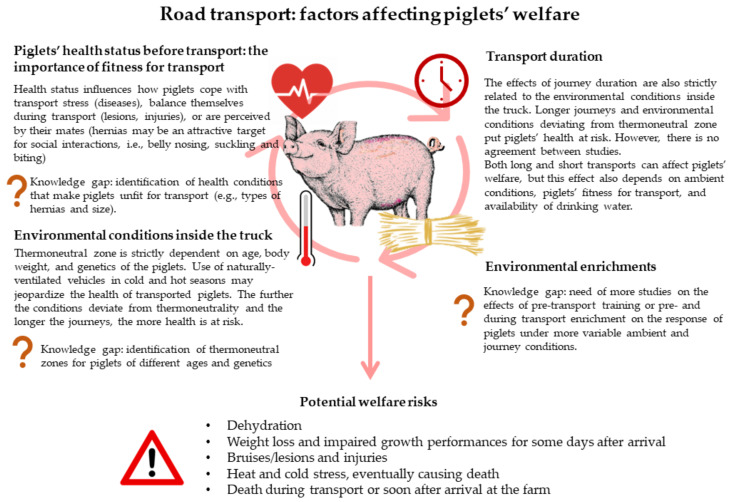
Summary of factors influencing the welfare of piglets during transport and on arrival. Knowledge gaps are also highlighted.

Figure 2 summarizes the major factors affecting the welfare of piglets during transport, highlighting the consensus between studies and the gaps in knowledge arising from the existing literature review.

## 6. Conclusions

Among pig categories, piglets are the most transported pig type after slaughter pigs. More than 14 million piglets are transported from northern European countries to other EU member states every year [79]. In Canada, approximately 18 million weaned piglets are transported domestically (J. Clark, Canadian Pork Council, personal communication) as well as approximately 400,000 newly weaned piglets (<7 kg) have been exported from Western Canada alone to the USA in 2020 [80]. Despite this extended trading activity, it is surprising to acknowledge the considerable gap in the scientific literature on the effects of transport and related factors on piglets’ welfare and post-transport growth performances.

Weaning is one of the most sensitive phases in pig farming as it represents a phase of change in both the diet and the environment in which newly-weaned piglets live. Since transport is combined with the beginning of this phase, it is reasonable to assume that at least part of the data concerning morbidity and mortality of pigs during and immediately after weaning may be due to the additive effect of these events. This association of stressors does not ease the evaluation of the contribution of feed and water deprivation to piglets’ response to weaning and transport stress. The different ages at weaning (from 17 to about 30 days of age), and therefore in liveweights, may help explain the significant discrepancy in the results between studies. Furthermore, although the results showing market weight response to transport stress, for which a much larger body of research has been carried out, cannot be representative of the interpretation of piglets’ welfare during transport, it can be generally concluded that, similarly to adult pigs, piglets are particularly vulnerable to heat stress and both long and short transport duration, with related effects being confounded by genetic background, fitness condition before transport, ambient conditions [4], transport vehicle used and lack of drinking water.

Future studies should consider morbidity, postures, body temperature and mortality rates in piglets during transport and post-transport growth performance focusing on factors, such as vehicle design, within truck stocking density and environmental factors (i.e., temperature, RH, noxious gases concentrations, noise and vibrations), piglet genetic background, and weaning age. This information is vital to develop science-based recommendations for optimal minimum transport conditions ensuring the physical comfort of weaned pigs during transport. Furthermore, given the close relationship between stocking density and thermal needs of piglets during transport, the new EU recommendations based on calculated allometric equations for floor space density must be validated by scientific research in order to make sure they are adjusted to the piglets’ thermal needs under variable external and within truck environmental conditions during the journey. 

## Data Availability

Not applicable.
